# Supplementary material for: Efficacy and tolerability of bevacizumab in patients with severe Covid-19
Source: Nat Commun. 2021 Feb 5;12:814. doi: 10.1038/s41467-021-21085-8 (PMC7864918; doi:10.1038/s41467-021-21085-8)
Supplement: Supplementary file 1 — Supplementary Information [file 41467_2021_21085_MOESM1_ESM.pdf]

SUPPLEMENTARY FILES

Supplementary table 1. Laboratory tests*

Laboratory tests	*n*	Baseline median [IQR]	Day 7 median [IQR]	*p* value
White blood cell count, $\times 10^9/L$	14	8.9 (6.6, 11.7)	9.5 (6.9, 11.1)	0.296
Neutrophil count, $\times 10^9/L$	14	6.3 (5.3, 9.7)	7.4 (3.8, 8.6)	0.542
Lymphocyte count, $\times 10^9/L$	14	0.9 (0.8, 1.2)	1.4 (1.0, 2.0)	0.005
Platelet count, $\times 10^9/L$	14	268.5 (234.0, 392.0)	312.0 (285.0, 386.0)	0.715
Red blood cell count, $\times 10^{12}/L$	14	4.6 (4.0, 4.7)	4.3 (3.8, 4.7)	0.199
Hemoglobin, g/*dL*	14	13.3 (12.8, 14.0)	12.7 (11.8, 13.6)	0.187
C-reactive protein, mg/*L*	9	69.4 (60.7, 139.9)	5.4 (2.5, 27.3)	0.020
D-dimer, ug/*dL*	9	475.0 (361.0, 965.0)	544.0 (214.0, 1062.0)	0.570
Activated partial thromboplastin time(*s*)	7	31.0 (27.0-37.0)	33.0 (26.3-35.0)	0.438
Fibrinogen(mg/*dL*)	5	694.0 (284.0-817.0)	419.0 (343.0-568.0)	0.438

*Laboratory values of white blood cell count, neutrophil count, lymphocyte count, platelet count, red blood cell count, and hemoglobin on baseline and day 7 after bevacizumab treatment were based on available data from 10 Italian patients and 4 Chinese patients. C-reactive protein and D-dimer data were collected from 8 Italian patients and 1 Chinese patient. IQR=interquartile range. Wilcoxon matched-pairs signed-rank test were used to calculate *p* values. $P < 0.05$ for two-tailed hypothesis tests was considered statistically significant. Source data are provided as a Source Data file.

Supplementary table 2. Comparison to other therapeutic studies in patients with severe Covid-19[1-4]

	Study	Study type	Country	Public-ation	Sample size	Treatment	Oxygen-support days [a,b]	Oxygen-support or clinical improvement Rate	Oxygen-support or clinical improvement Days [b]	Disch-arge rate [d]	Mort-ality [d]
1	J Pang et al.	Single-arm trial	China, Italy	Not yet	26	Bevacizumab	9 (5,18)	92% [c]	28	65%	0%
					26	control	20 (16,28)	62% [c]	28	46%	19%
2	J Grein et al.	Single-arm trial	USA, Japan, Italy, et al.	N Engl J Med	53	Remdesivir	/	68% [c]	18 (13,23)	47%	13%
3	Y Wang et al.	RCT	China	Lancet	158	Remdesivir	19 (11,30)	65%	28	61%	14%
					78	placebo	21 (14,30)	58%	28	58%	13%
4	B Cao et al.	RCT	China	N Engl J Med	99	Lopinavir–Ritonavir	12 (9,16)	78.8%	28	/	19.2%
					100	Standard care	13 (6,16)	70.0%	28	/	25.0%
5	L Li et al.	RCT	China	JAMA	52	Convalescent Plasma	/	51.9%	28	51%	15.7%
					51	Control	/	43.1%	28	36%	24.0%

[a] Data are shown as median [IQR].

[b] Days from randomization or intervention.

[c] These data refer to rates of improvement of oxygen-support class.

[d] Discharge or mortality rates within the duration of 28 days.

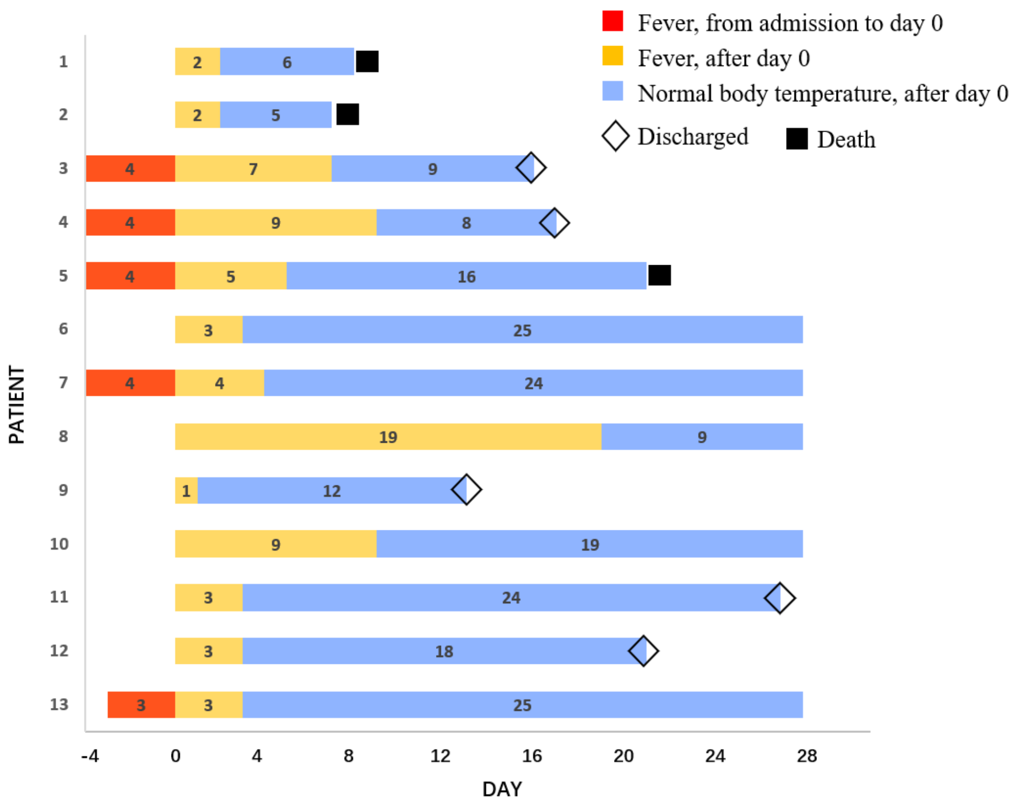

Supplementary Figure 1. Changes in fever symptom of individual control patients. Dynamic changes of fever status of 13 controls who had fever at day 0. Red, orange, and blue columns indicate the duration of fever or normal body temperature status. Diamonds represent discharge, and solid square symbols represent death.

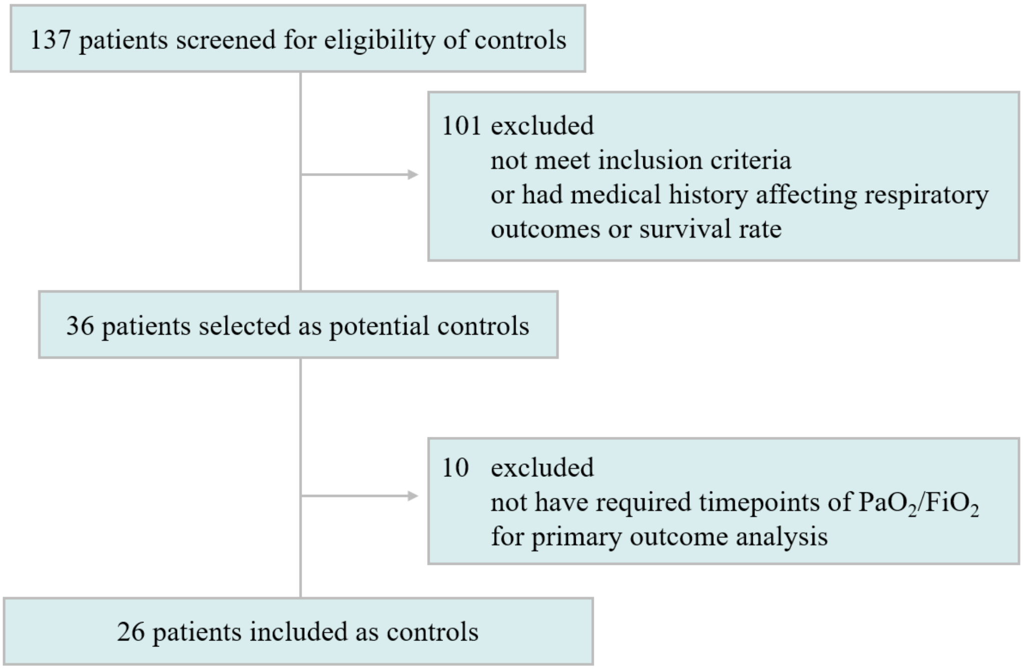

Supplementary Figure 2. CONSORT flow diagram of external controls.

PaO_2/FiO_2=partial arterial oxygen pressure to fraction of inspiration O_2 ratio

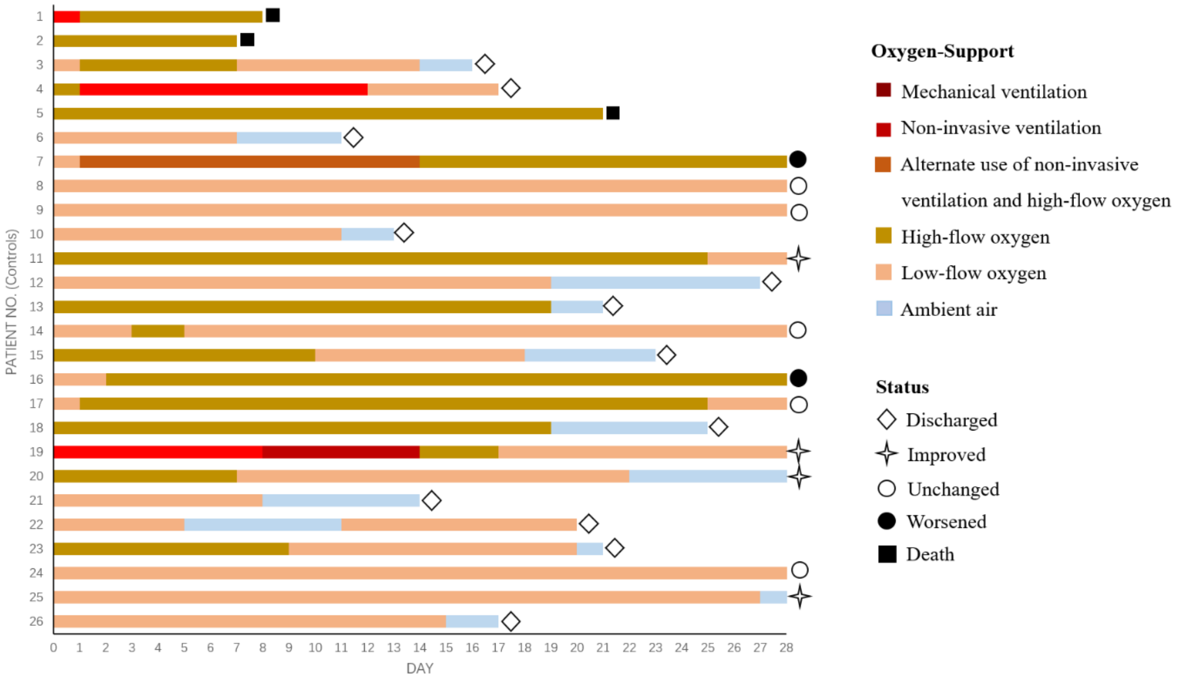

Supplementary Figure 3. Changes in oxygen-support status in individual external control patients. For each individual control patient, colored columns represent the oxygen-support status of the patient over time. Diamond symbols represent patients discharged from hospitals; star marks represent patients improved but not discharged; circle symbols show patients unchanged; solid circle symbols show patients worsened; solid square symbols show patients dead.

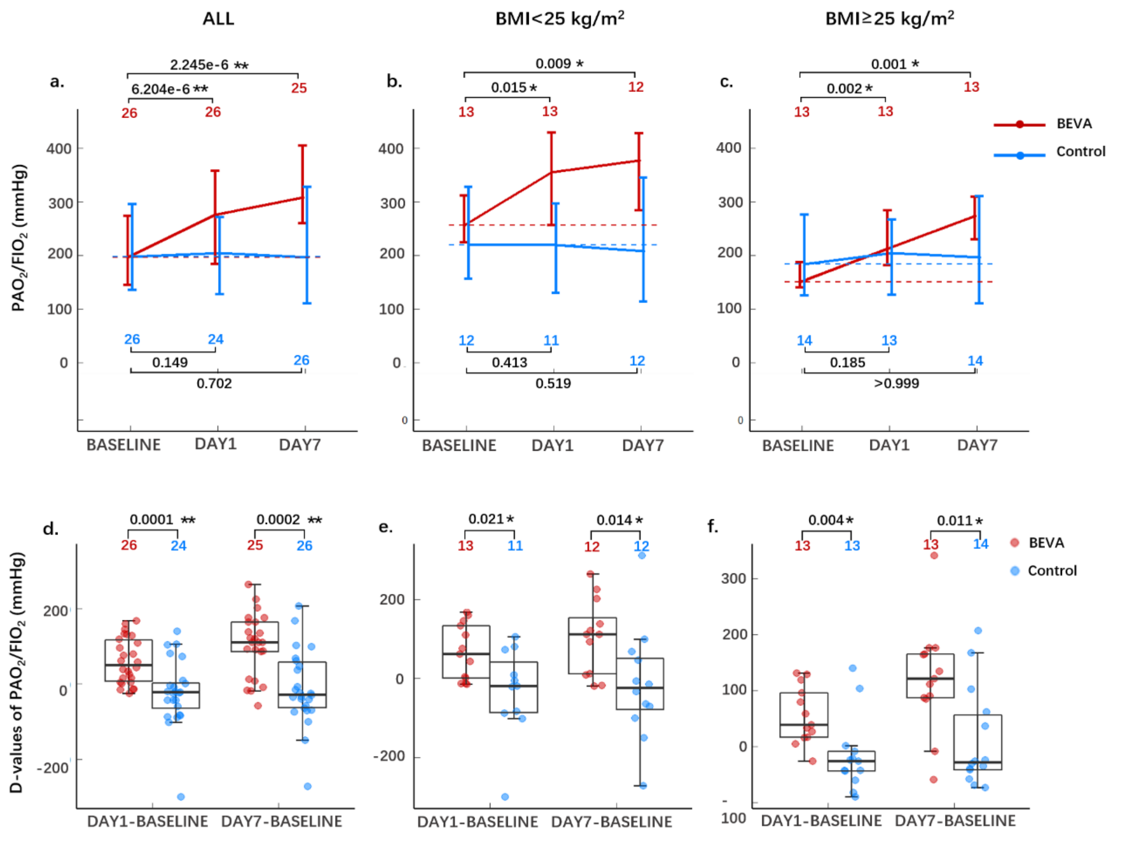

Supplementary Figure 4. Subgroup analysis by BMI (Body Mass Index) in relation to dynamic changes of PaO_2/FiO_2 and related comparisons. PaO_2/FiO_2 values in all patients (**a**), BMI < 25 kg/m^2 (**b**), and BMI ≥ 25 kg/m^2 (**c**) groups. The dashed lines represent the baselines; nodes in the lines represent the median values; the lower and upper values of the bar correspond to the 25th and 75th percentiles (Q1 and Q3); the length of the bar represents interquartile range (IQR); red and blue numbers correspond to the sample sizes (*n*); numbers on the horizontal lines represent *p* values. D-values (the difference values between day 1 or day 7 and baseline) of individual patients were utilized for the comparisons between BEVA (bevacizumab) and control groups of all patients (**d**), BMI < 25 kg/m^2 (**e**) and BMI ≥ 25 kg/m^2 (**f**) groups. The center lines of boxes represent median values; the lower and upper hinges represent Q1 and Q3, the range between which represents IQR; whiskers correspond to the highest or lowest values of non-outlier data (within 1.5 × IQR from the lower or upper hinges); numbers on the horizontal lines represent *p* values; red and blue numbers correspond to the sample sizes (*n*); the red and blue dots represent the data of individual patients. For (**a-c**), *p* values were calculated by Wilcoxon matched-pairs signed-rank test. For (**d-f**), *p* values were calculated by Wilcoxon signed-rank test. PaO_2/FiO_2=partial arterial oxygen

pressure to fraction of inspiration O_2 ratio. P <0.05 for two-tailed hypothesis tests was considered statistically significant. Source data are provided as a Source Data file.

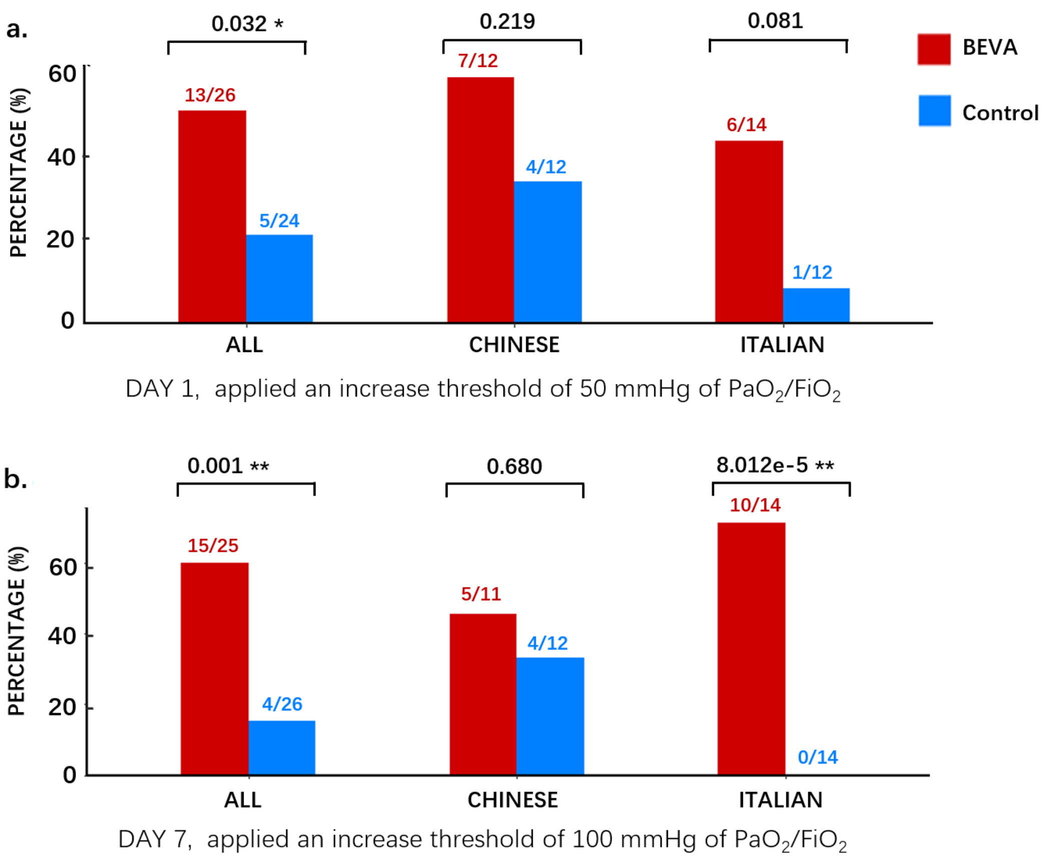

Supplementary Figure 5. Percentages of patients with increased PaO_2/FiO_2 values reaching certain thresholds. The increase thresholds of PaO_2/FiO_2 were set to 50 mmHg at day 1 (**a**) and 100 mmHg at day 7 (**b**). Numbers on top of each column represents quantity of patients; p values were calculated by Chi-square test and shown on the horizontal line. PaO_2/FiO_2=partial arterial oxygen pressure to fraction of inspiration O_2 ratio. BEVA=bevacizumab. P <0.05 for two-tailed hypothesis tests was considered statistically significant. Source data are provided as a Source Data file.

References:

1 Grein, J. et al. Compassionate Use of Remdesivir for Patients with Severe Covid-19. N. Engl. J. Med. 382, 2327-2336 (2020).

2 Wang, Y. et al. Remdesivir in adults with severe COVID-19: a randomised, double-blind, placebo-controlled, multicentre trial. Lancet 395, 1569-1578 (2020).

3 Cao, B. et al. A Trial of Lopinavir-Ritonavir in Adults Hospitalized with Severe Covid-19. N. Engl. J. Med. 382, 1787-1799 (2020).

4 Li, L. et al. Effect of Convalescent Plasma Therapy on Time to Clinical Improvement in Patients With Severe and Life-threatening COVID-19: A Randomized Clinical Trial. JAMA 324, 460-470 (2020).

Supplementary Note 1. Study Protocol

Efficacy and Safety of Bevacizumab in Severe Patients with Covid-19

（BEST Study）

Protocol

Version: Feb 14, 2020

1. BACKGROUND

Vascular endothelial growth factor (VEGF), also known as vascular permeability factor (VPF), is a subfamily of growth factors. Hypoxia induces VEGF expression through activation of the Prolyl hydroxylases (PHD)-hypoxia inducible factor (HIF)-1 pathway, which upregulates VEGF expression through transcription activation. Patients with severe Covid-19 suffer from severe hypoxia, and VEGF levels in patients with severe Covid-19 are markedly elevated. VEGF is a potent vascular permeability factor that induces vascular leakiness in Covid-19-infected lung tissues, resulting in plasma extravasation and pulmonary edema, which further increases tissue hypoxia. Bevacizumab is a recombinant humanized monoclonal antibody. As an anti-VEGF medication, it has been prevalently utilized in oncotherapy since 2004, with considerable reliability and clinical safety.

Supportive clinical and nonclinical profile for the use of bevacizumab in severe patients with Covid-19 includes:

- Patients with severe Covid-19 suffer from severe hypoxia. Hypoxia is known to significantly induce VEGF expression through activation of the HIF-1 pathway[1,2].

- VEGF levels in patients with severe Covid-19 are markedly elevated[3]. VEGF contributes to increased vascular permeability and pulmonary edema[4,5].

- Pulmonary edema frequently presents in Covid-19 patients. Autopsy of Covid-19 patients shows excessive extravasates in alveoli of the infected lungs[6].

- Overreactive inflammatory response happens in Covid-19. VEGF is known to enhance inflammation in the lung[7].

- In a mouse model of Acute Respiratory Distress Syndrome (ARDS), Watanabe M et al. used intratracheal adenovirus (Ad)-mediated overexpression of human vascular endothelial growth factor in mouse lung to induce alveolar permeability and consequent pulmonary edema, and demonstrated that bevacizumab suppressed vascular endothelial growth factor-induced high-permeability pulmonary edema.

- David R et al found plasma VEGF was significantly elevated in patients with ARDS compared with at-risk patients. In vitro study showed, the peripheral blood mononuclear cells from patients with ARDS produced remarkably more VEGF in vitro than at-risk patients, and VEGF inhibitors significantly ameliorated the permeability of human lung epithelial cells.

- Bevacizumab has been widely used in cancer treatment since 2004, with considerable reliability and clinical safety. It was reported that bevacizumab rapidly reduced macular edema and contributed to improve visual acuity in patients with wet age-related macular degeneration[8].

2. PATIENT ENROLLMENT

2.1 Inclusion Criteria

1) Age: 18-80 years old, both genders.

2) Confirmed COVID-19 diagnosis. A confirmed case is based on epidemiological history (including cluster transmission), and results of SARS-CoV-2 nucleic acid detection.

3) Respiratory distress, RR ⩾ 30 times/min; SpO2 ⩽ 93% at rest; or PaO2/FiO2 ratio >100mmHg and ⩽ 300mmHg (1mmHg = 0.133kPa).

4) Pulmonary imaging shows diffuse exudative lesions.

2.2 Exclusion Criteria

1) Patients with severe hepatic dysfunction (Child-Pugh score ≥ C or aspartate aminotransferase level > 5 times the upper reference limit, URL);

2) Patients with severe renal dysfunction (estimated glomerular filtration rate ≤ 30 mL/min/1.73 m^2) or who required continuous renal replacement therapy, haemodialysis, or peritoneal dialysis;

3) Patients with uncontrolled hypertension (sitting systolic blood pressure > 160 mmHg or diastolic blood pressure >100 mmHg) or a history of hypertension crisis or hypertensive encephalopathy;

4) Patients with poorly controlled heart diseases, such as New York Heart Association class II or higher cardiac insufficiency, unstable angina pectoris, myocardial infarction within 1 year before enrollment, or supraventricular or ventricular arrhythmia needing treatment or intervention;

5) Patients with hereditary bleeding tendency or coagulopathy, and patients who received full-dose anticoagulant or thrombolytic therapy within 10 days before enrollment, or non-steroidal anti-inflammatory drugs with platelet suppression within 10 days before enrollment (except those who used small doses of aspirin [≤325 mg/day] for preventive use);

6) Patients with thrombosis within 6 months before enrollment, patients who had experienced arterial/venous thromboembolic events, such as ischemic stroke, transient ischemic attack, deep venous thrombosis, or pulmonary embolism within 1 year prior to trial enrollment, and patients with severe vascular disease (including aneurysms or arterial thrombosis requiring surgery) within 6 months before trial enrollment;

7) Patients with unhealed wounds, active gastric ulcers, or fractures; patients with gastrointestinal perforation, gastrointestinal fistula, abdominal abscess, or visceral fistula formation within 6 months before trial enrollment;

8) Patients who had undergone major surgery (including preoperative chest biopsy) or received major trauma (such as a fracture) within 28 days before enrollment; patients who might need surgery during the trial;

9) Patients with severe, active bleeding such as haemoptysis, gastrointestinal bleeding, central nervous system bleeding, and epistaxis within 1 month before trial enrollment;

10) Patients with malignant tumours within 5 years before trial enrollment;

11) Patients allergic to bevacizumab or its components;

12) Patients with untreated active hepatitis or HIV-positive patients; pregnant and lactating women and those planning to get pregnant;

13) Patients who participated in other clinical trials or not considered suitable for this trial by the researchers; or 14) patients who did not provide signed informed consent.

3. DRUG DESCRIPTION

3.1 Dosage

Bevacizumab at a dose range of 5-15 mg/kg is routinely used in oncology. A single dose of 500mg (about 7.5 mg/kg) used in this study was within the lower range.

3.2 Route of Administration

A single dose of bevacizumab 500mg + saline 100ml is administered intravenously in no less than 90 min under electrocardiogram monitoring.

3.3 Pharmacokinetics of Bevacizumab

The pharmacokinetic profile of bevacizumab is provided according to the drug manuscript as follows:

The pharmacokinetic profile of bevacizumab was assessed using an assay that measured total serum bevacizumab concentrations (i.e., the assay did not distinguish between free bevacizumab and bevacizumab bound to VEGF ligand). Based on a population pharmacokinetic analysis of 491 patients who received 1 to 20 mg/kg of bevacizumab every week, every 2 weeks, or every 3 weeks, Bevacizumab pharmacokinetics are linear and the predicted time to reach more than 90% of steady state concentration is 84 days. The accumulation ratio following a dose of 10 mg/kg once every 2 weeks is 2.8. Population simulations of bevacizumab exposures provide a median trough concentration of 80.3 mcg/mL on Day 84 (10th, 90th percentile: 45, 128) following a dose of 5 mg/kg once every two weeks.

Distribution: The mean (% coefficient of variation [CV%]) central volume of distribution is 2.9 (22%) L.

Elimination: The mean (CV%) clearance is 0.23 (33) L/day. The estimated half-life is 20 days (11 to 50 days).

Specific Populations: The clearance of Bevacizumab varied by body weight, and sex. After correcting for body weight, males had a higher Bevacizumab clearance (0.26 L/day vs. 0.21 L/day) and a larger central volume of distribution (3.2 L vs. 2.7 L) than females.

4. CLINICAL PROCEDURES, ARTERIAL BLOOD GAS ANALYSIS, CHEST RADIOLOGICAL IMAGING, OXYGEN SUPPORT AND LABORATORY TESTS

4.1 Informed Consent

The researcher informs the patient about the trial's detailed information. After signing the informed consents, the patients will be assessed if they are eligible for this study.

4.2 Prior to Drug Administration

Physical examination and vital signs assessment will be conducted. Arterial blood gas (ABG) assay will be performed within 24 hours prior to bevacizumab administration. Chest CT or chest X-ray will be performed within 48 hours prior to bevacizumab treatment. Patient’s demographic information, medical history, diagnosis of Covid-19, current medication, oxygen-support status and laboratory tests will be collected.

4.2 ABG assay

ABG assay will be performed on day 1 and day 7 post-bevacizumab administration.

4.3 Chest Radiological imaging

Chest CT scanning will be performed on day 7 (±24hrs) post-bevacizumab administration. Take consideration that the medical resources during the pandemic situation are limited, alternative Chest X-ray will be acceptable, which will be performed on day 3 and day 7 (±24hrs) post-bevacizumab administration.

4.4 Oxygen-support status

Oxygen-support status including extracorporeal membrane oxygenation, mechanical support, non-invasive support, an intermediate status of alternation of non-invasive support and high-flow oxygen, high-flow oxygen, and low-flow oxygen will be recorded daily from the day of bevacizumab administration to day 28. Venturi mask is regarded as low-flow oxygen. Reservoir with oxygen flow⩾10 L/min is considered as high-flow oxygen. Oxygen inhalation through nasal catheter or mask regardless of the oxygen flow is identified as low-flow oxygen.

4.5 Others

It is recommended that use of corticosteroids be avoided, if possible.

Physical examination and vital signs will be performed daily.

Laboratory tests including blood routine, hepatic and renal function tests, CRP and coagulation factors) will be performed at baseline (within 48 hours prior to bevacizumab administration) and day 7 (±24hrs) post-bevacizumab administration.

5. BENEFIT-RISK ASSESSMENT

No investigational drugs with proven clinical efficacy for severe Covid-19 is available. Dyspnoea and inflammatory pulmonary edema present in almost all patients with severe Covid-19 and needs oxygen-support and long hospital stay. The levels of VEGF, a potent vascular permeability factor that induces vascular leakiness, are markedly elevated in Covid-19 patients. VEGF also significantly participates in lung inflammation. Blocking VEGF and the VEGFR-mediated signalling by would improve oxygen perfusion and anti-inflammatory response and alleviate clinical symptoms in patients with severe Covid-19. Bevacizumab would address the urgent need for developing effective drugs in this serious pandemic situation.

The pharmacokinetic profile of bevacizumab has been assessed using an assay that

measured total serum bevacizumab concentrations (i.e., the assay did not distinguish between free bevacizumab and bevacizumab bound to VEGF ligand) as mentioned above. The rate of infusion is based on the patient's tolerance, and the first intravenous infusion needs to last 90 minutes. In the dose range of 1 to 10 mg/kg, the pharmacokinetics of bevacizumab show a linear relationship. The metabolism and elimination of bevacizumab is similar to endogenous IgG, which is mainly through proteolytic catabolism of human body including endothelial cells, instead of the kidney and the liver. The binding of IgG to FcRn protects it from being metabolized by cells and has a long terminal half-life. The clearance of Bevacizumab varies by body weight, and sex. The estimated elimination half-life of a typical female patient is 18 days, and a typical male patient is 20 days. The pharmacokinetics of bevacizumab doses not differ significantly between different ages in adults. Pharmacokinetic studies of bevacizumab in patients with kidney injury or liver damage have not been conducted because the kidney or the liver are not the main organ for bevacizumab metabolism or excretion.

Over the past years, several clinical trials of bevacizumab in the treatment of different malignant tumors have been carried out, most of which are combined with chemotherapy drugs. This paragraph describes the safety results obtained from the clinical trial population of approximately 5,500 patients. The most serious adverse reactions are: gastrointestinal perforation, bleeding (mostly pulmonary hemorrhage and hemoptysis in patients with non-small cell lung cancer) and arterial thromboembolism. The most frequent adverse reactions include hypertension, fatigue, diarrhea and abdominal pain. The analysis of clinical safety data suggests that the incidence of hypertension and proteinuria may be dose-dependent when receiving bevacizumab.

In pre-clinical animal experiments, the incidence and severity of bevacizumab's toxic and side effects are related to the dose, and can be partially recovered after drug withdrawal, which includes growth plate dysplasia, reduced wound healing ability, and possibly affecting fertility, the dose is 0.4-20 times the recommended dose for human use. In trials tested by human volunteers, the highest dose (20 mg/kg body weight, given every 2 weeks, intravenous infusion) may cause severe migraine in some patients.

6. SAFETY MONITORING

Possible adverse events related to this study include: hypertension, nausea, vomiting, diarrhea, abdominal pain, neutropenia, leukocytopenia, thrombocytopenia, lymphopenia, anemia, headache, increased tearing, epistaxis, stomatitis, exfoliative dermatitis, proteinuria, gastrointestinal perforation, bleeding and arterial thromboembolism, etc. Adverse events will be monitored and adjudicated by the Safety Monitoring Committee. All the adverse events will be handled timely with proper medical treatment to avoid further damage.

7. REFERENCES

1) Liu Y, Cox SR, Morita T, Kourembanas S. Hypoxia regulates vascular endothelial growth factor gene expression in endothelial cells. Identification of a 5' enhancer. Circ Res 1995;77:638-43.
2) Marti HH, Risau W. Systemic hypoxia changes the organ-specific distribution of vascular endothelial growth factor and its receptors. Proc Natl Acad Sci U S A 1998;95:15809-14.
3) Huang C, Wang Y, Li X, et al. Clinical features of patients infected with 2019 novel coronavirus in Wuhan, China. Lancet 2020;395:497-506.
4) Thickett DR, Armstrong L, Christie SJ, Millar AB. Vascular endothelial growth factor may contribute to increased vascular permeability in acute respiratory distress syndrome. Am J Respir Crit Care Med 2001;164:1601-5.
5) Zhang Z, Wu Z, Xu Y, Lu D, Zhang S. Vascular endothelial growth factor increased the permeability of respiratory barrier in acute respiratory distress syndrome model in mice. Biomed Pharmacother 2019;109:2434-40.
6) Liu Q, Wang RS, Qu GQ, et al. Gross examination report of a COVID-19 death autopsy. Fa Yi Xue Za Zhi 2020;36:21-3.
7) Lee CG, Link H, Baluk P, et al. Vascular endothelial growth factor (VEGF) induces remodeling and enhances TH2-mediated sensitization and inflammation in the lung. Nat Med 2004;10:1095-103.
8) Georgalas I, Papaconstantinou D, Paraskevopoulos T, Koutsandrea C. Bevacizumab must be specially prepared for intraocular use. BMJ 2013;347:f5032.

8. APPENDICES

Case Report Form

1. Basic Information

1.1 Basic Information
1.1.1 Gender: □Male □Female<br>1.1.2 Date of birth: _ _ / _ _ / _ _ _ _<br>1.1.3 Height: ______cm Weight: ______kg
**1.2 Medical History**
1.2.1 Hypertension : □No (Yes<br>1.2.2 Diabetes: □No □Yes<br>1.2.3 COPD: □No □Yes<br>1.2.4 Acute Coronary Syndrome: □No □Yes<br>1.2.5 Myocardial Infarction: □No □Yes<br>1.2.6 Heart Failure: □No □Yes<br>1.2.7 Arrhythmia: (No □Yes<br>1.2.8 Cerebral Infarction: □No □Yes<br>1.2.9 Cerebral Hemorrhage: □No □Yes<br>1.2.10 Heptic Insufficiency: (No □Yes, name of disease______________<br>1.2.11 Renal Insufficiency: □No □Yes, name of disease______________<br>1.2.12 Hemopathy: □No □Yes: __________<br>1.2.13 Tumor: (No □Yes, benign, name: __________<br>□Yes, malignancy, name: __________

2. Onset Information

2.1 Onset date: _ _ / _ _ / _ _ _ _   _ _ : _ _   (MM / DD / YYYY   HH : MM)

2.2 Admission date: _ _ / _ _ / _ _ _ _   _ _ : _ _

Our hospital is your first visit hospital: □No   □Yes

If No, please fill in the first visit hospital name: _____________

date of first visit: _ _ / _ _ / _ _ _ _

2.3 Clinical signs and symptoms:

□Fever: Tmax________□   ☐Weak

□Dyspnea   □Chest distress   □Shortness of breath   □Dry cough   □Expectoration

□Nausea   □Vomit   □Diarrhea   ☐Stomachache

□Muscular soreness   □Joint sore   □Headache   □Pharyngalgia

□Conjunctival congestion   □Others: ____________

2.4 Complications: ☐Yes   □No

If Yes, please fill in the forms below (multiple choices):

□ALI / ARDS   □Secondary bacterial pneumonia

□Sepsis   □Acute myocardial injury   □Acute kidney injury

□Meningitis   □Encephalitis   □Epilepsy

□Others: ___________

3. Visit 1 Evaluation Prior Drug Administration

3.1 Oxygenation index and vital signs (within 24h prior drug administration)

3.1.1 PaO_2: _______mmHg Oxygen flow: _______ml/L SpO_2: ______%

3.1.2 Temperature: ______□ Pulse: ______beats/min

Respiration: ______breaths/min Blood pressure (supine): _____/_____mmHg

3.2 Blood Tests (within 48h before drug administration)

Test	Value
CRP (mg/L)	
WBC (×10^9/L)	
Neutrophils (×10^9/L)	
Lymphocyte (×10^9/L)	
Platelet (×10^9/L)	
HGB (g/L)	
ALT (U/L)	
AST (U/L)	
BUN	(□mmol/L □mg/dL)
Cr	(□mmol/L □mg/dL)

3.3 Chest CT (within 48h prior drug administration)

□Chest CT has been performed within 48h prior study drug administration

□Chest CT can not be performed. Chest X ray has been performed within 48h prior study drug administration.

4. Oxygen-support status（record daily）

a. Extracorporeal Membrane Oxygenator (ECMO); b. Mechanical ventilation;

c. non-invasive ventilation; d. alternation of non-invasive ventilation and high-flow oxygen; e. high-flow oxygen; f. low-flow oxygen.

Day 0 drug use		Day 15	
Day 1		Day 16	
Day 2		Day 17	
Day 3		Day 18	
Day 4		Day 19	
Day 5		Day 20	
Day 6		Day 21	
Day 7		Day 22	
Day 8		Day 23	
Day 9		Day 24	
Day 10		Day 25	
Day 11		Day 26	
Day 12		Day 27	
Day 13		Day 28	
Day 14			

5. Visit 2 (Day 1 _ _ / _ _ / _ _ _ _)

**5.1 Patient status**
□Die If Died, please fill in the cause of death: _______________
□Alive If Alive, please fill in the forms below
**5.2 Oxygenation index and vital signs**
5.2.1 PaO_2: _______mmHg Oxygen flow: _______ml/L SpO_2: ______%
5.2.2 Temperature: ______□ Pluse: ______beats/min
Respiration: ______breaths/min Blood pressure (supine): _____/_____mmHg

6. Visit 3 (Day 3 _ _ / _ _ / _ _ _ _)

**Chest X-ray (Day 3)**
□Chest CT can not be performed. Chest X ray has been performed 3d after drug administration
□ Other condition, specify: ______

7. Visit 4 (Day7 _ _ / _ _ / _ _ _ _)

7.1 Patient status

□Die If Died, please fill in the cause of death: _______________

□Alive If Alive, please fill in the forms below

7.2 Oxygenation index and vital signs

7.2.1 PaO_2: _______mmHg Oxygen flow: _______ml/L SpO_2: ______%

7.2.2 Temperature: ______□ Pluse: ______beats/min

Respiration: ______breaths/min Blood pressure (supine): _____/_____mmHg

7.3 Blood Tests

Test	Value
CRP (mg/L)	
WBC ($\times 10^9$/L)	
Neutrophils ($\times 10^9$/L)	
Lymphocyte ($\times 10^9$/L)	
Platelet ($\times 10^9$/L)	
HGB (g/L)	
ALT (U/L)	
AST (U/L)	
BUN	(□mmol/L □mg/dL)
Cr	(□mmol/L □mg/dL)

7.4 Chest CT

□Chest CT has been performed within 48h prior study drug administration

□Chest CT can not be performed. Chest X ray has been performed within 48h prior study drug administration.

8. Visit 5 (Day 28 _ _ / _ _ / _ _ _ _)

**8.1 Patient status**
□Die   If Died, please fill in the cause of death: ______________<br>□Alive   If Alive, please fill in the forms below
**8.2 ICU and Hospital Stays**
8.2.1 Admitted to ICU：☐ No ☐ Yes   *If "Yes", please specify：*<br>Duration: _ _ / _ _ / _ _ _ _ _ _ : _ _ to _ _ / _ _ / _ _ _ _ _ _ : _ _<br>Still in ICU at Day 28：☐ No ☐ Yes<br>8.2.2 Discharge Date: _ _ / _ _ / _ _ _ _ _ _ ☐Still stay in hospital at Day 28<br>☐Not applicable

Appendix Form 1 Concomitant Medications

Concomitant medications since informed consent:

Drug Name	Indications	Dose per day	Reason for drug use	Frequency	Dose Unit	Route	Start Date	End Date
						|_|	_ _/ _ _/ _ _ _ _	☐Ongoing ☐End _ _/ _ _/ _ _ _ _
						|_|	_ _/ _ _/ _ _ _ _	☐Ongoing ☐End _ _/ _ _/ _ _ _ _
						|_|	_ _/ _ _/ _ _ _ _	☐Ongoing ☐End _ _/ _ _/ _ _ _ _
						|_|	_ _/ _ _/ _ _ _ _	☐Ongoing ☐End _ _/ _ _/ _ _ _ _
						|_|	_ _/ _ _/ _ _ _ _	☐Ongoing ☐End _ _/ _ _/ _ _ _ _
						|_|	_ _/ _ _/ _ _ _ _	☐Ongoing ☐End _ _/ _ _/ _ _ _ _
						|_|	_ _/ _ _/ _ _ _ _	☐Ongoing ☐End _ _/ _ _/ _ _ _ _
						|_|	_ _/ _ _/ _ _ _ _	☐Ongoing ☐End _ _/ _ _/ _ _ _ _
						|_|	_ _/ _ _/ _ _ _ _	☐Ongoing ☐End _ _/ _ _/ _ _ _ _
						|_|	_ _/ _ _/ _ _ _ _	☐Ongoing ☐End _ _/ _ _/ _ _ _ _
						|_|	_ _/ _ _/ _ _ _ _	☐Ongoing ☐End _ _/ _ _/ _ _ _ _
						|_|	_ _/ _ _/ _ _ _ _	☐Ongoing ☐End _ _/ _ _/ _ _ _ _
						|_|	_ _/ _ _/ _ _ _ _	☐Ongoing ☐End _ _/ _ _/ _ _ _ _
						|_|	_ _/ _ _/ _ _ _ _	☐Ongoing ☐End _ _/ _ _/ _ _ _ _

Routes:1.Oral administration 2. Intravenous infusion 3. Local external use 4. Nasal feeding 5. Subcutaneous injection 6. Intramuscular injection 7. Ear drops 8. Eye drops 9. Hypoglossal 10. Inhalation 11. Nasal penetration 12. Transdermal 13. Transvaginal 14. Transrectal 15. Epidural 16. Intrathecal 17. Intra-articular 18. Periarticular 19. Intraperitoneal 20. Intradermal 21. Intra-arterial 22. Others.

Comments： This form can be duplicated if necessary

Signature of researcher： ________________ **Date：** |__|__|/|__|__|/|__|__|__|__|

Appendix Form 2 ADVERSE EVENTS

Whether the subject has experienced any adverse event during the study:

☐ No ☐ Yes *If "No", please check the box:* ☐ Not Applicable

If "Yes", please specify below:

<table>
<tr><td colspan="3">The Adverse Event:</td></tr>
<tr><td colspan="3">Description of the Adverse Event：</td></tr>
<tr><td colspan="3">Duration: _ _ / _ _ / _ _ _ _ _ _ : _ _ to _ _ / _ _ / _ _ _ _ _ _ : _ _</td></tr>
<tr><td colspan="3">Severity: ☐ Mild ☐ Moderate ☐ Severe</td></tr>
<tr><td>Relationship to the Study Drug:</td><td>☐ Definite
☐ Unlikely</td><td>☐ Probable ☐ Possible
☐ Unrelated</td></tr>
<tr><td>Effect on the dose of the Study Drug:</td><td>☐ None
☐ Interrupted</td><td>☐ Dose increased ☐ Dose Reduced
☐ Withdrawn</td></tr>
<tr><td>Whether to take action on AE:</td><td>☐ No</td><td>☐ Yes (If "Yes", please specify the concomitant medication and treatment)</td></tr>
<tr><td>Outcome of AE:</td><td>☐ Recovered
☐ Unknown</td><td>☐ Recovering ☐ Not Recovered
☐ Unclear______</td></tr>
<tr><td colspan="3">The subject withdrawn from the trial due to AE? ……………………☐ No ☐ Yes</td></tr>
<tr><td colspan="3">Whether it is a SAE? ………………………☐ No ☐ Yes</td></tr>
<tr><td colspan="3">Please Specify Below (Only for SAE)</td></tr>
<tr><td colspan="3">Complete the SAE form? …………… ☐ Yes ☐ No
Whether the SAE has been reported to relevant parties within 24 hours? …… ☐ Yes ☐ No</td></tr>
<tr><td>Reasons of being reported as SAE：</td><td>☐ Fatal
☐ Hospitalization or Prolonged Hospitalization
☐ Congenital Anomaly/ Birth Defect</td><td>☐ Life Threating
☐ Persistent or Severe Disability/Incapacity
☐ Important medical event</td></tr>
</table>

Note：This form can be duplicated if necessary. SAE Form should be submitted to Ethics Committee.

Signature of researcher： _______________ **Date：** |__|__|/|__|__|/|__|__|__|__|

Appendix Form 3 Serious adverse event

Whether the subject has experienced SAE since the informed consent was signed?

☐ No ☐ Yes *If "Yes", please specify below:*

SAE report must be summitted to Ethics Committee, sponsor (or CRO) and the study site by fax or telephone within 24 hours

<table>
<tr><th colspan="8">Serious Adverse Event Report Form</th></tr>
<tr><td colspan="2">Type of Report</td><td colspan="5">☐ First Report ☐Follow-up Report ☐ Summary Report</td><td>Report Time:</td></tr>
<tr><td colspan="2">Name of the Hospital and Specialty</td><td colspan="5"></td><td>Tel:</td></tr>
<tr><td colspan="2">Sponsor Name</td><td colspan="5"></td><td>Tel:</td></tr>
<tr><td colspan="2" rowspan="2">Drug Name</td><td colspan="6">Chinese Name:</td></tr>
<tr><td colspan="6">English Name:</td></tr>
<tr><td colspan="2">Register Types and Dosage Form of the Drug</td><td colspan="6">Types： ☐ Herbs ☐ Chemicals ☐Biologics for Therapy ☐ Biologics for prevention ☐ Others________________
Register Types: ________________ Dosage Form: ____________</td></tr>
<tr><td colspan="2">Category of Clinical Trial</td><td colspan="4">☐Phase □ ☐Phase □ ☐ Phase □ ☐Phase □
☐ Bioequivalence study ☐ Clinical Verification</td><td colspan="2">Indications of Clinical Trial：</td></tr>
<tr><td rowspan="2">Baseline Characteristics of the Subject</td><td>Initial of Names:</td><td colspan="2">Birth Date:</td><td>Gender:
☐ Male ☐ Female</td><td colspan="2">Height(cm):</td><td>Weight(Kg)：</td></tr>
<tr><td colspan="7">Concomitant Diseases and Medications： ☐ Yes ☐ No
1. Disease： __________ Mediaction： __________ Route and Dose： __________
2. Disease： __________ Medication： __________ Route and Dose： __________
3. Disease： __________ Medication： __________ Route and Dose： __________</td></tr>
<tr><td colspan="3">SAE Term (Diagnosis)</td><td colspan="5"></td></tr>
</table>

Category of the SAE	☐ Death: _ _ / _ _ / _ _ _ _<br>☐ Hospitalization ☐ Prolonged Hospitalization ☐ Disability ☐Incapacity<br>☐Congenital Anomaly ☐Life-Threating ☐Others_______
SAE Onset Date: _ _ / _ _ / _ _ _ _	Date subject Reported: _ _ / _ _ / _ _ _ _
Action to the Study Agent:	☐Continued ☐Dose Reduced ☐Suspension and continued later ☐Withdrawn ☐Interrupted
SAE Outcome	☐Symptoms disappear(Sequelae ☐Yes ☐No) ☐Symptoms last ☐Death
Relationship of the SAE to Study Drugs	☐ Definitely yes ☐ Probably yes ☐ Maybe ☐ Probably no ☐ Definitely no
SAE Reports	Domestic：☐Yes ☐No ☐Unknown；<br>Abroad：☐Yes ☐No ☐Unknown
What medications or other steps were taken to treat the SAE?	

Reporting orgnization： Position/professional title of reporter：

Signature of reporter：

Note：This form can be duplicated if necessary.

Signature of researcher： _______________ **Date：** |__|__|/|__|__|/|__|__|__|__|
