# Supplementary material for: Efficacy and tolerability of bevacizumab in patients with severe Covid-19
Source: Nat Commun. 2021 Feb 5;12:814. doi: 10.1038/s41467-021-21085-8 (PMC7864918; doi:10.1038/s41467-021-21085-8)
Supplement: Supplementary file 3 — Reporting Summary [file 41467_2021_21085_MOESM3_ESM.pdf]

nature research

Corresponding author(s): Yihai Cao, Yuguo Chen

Last updated by author(s): Oct 19, 2020

Reporting Summary

Nature Research wishes to improve the reproducibility of the work that we publish. This form provides structure for consistency and transparency in reporting. For further information on Nature Research policies, see our Editorial Policies and the Editorial Policy Checklist.

Statistics

For all statistical analyses, confirm that the following items are present in the figure legend, table legend, main text, or Methods section.

n/a	Confirmed	
☐	☒	The exact sample size (*n*) for each experimental group/condition, given as a discrete number and unit of measurement
☐	☒	A statement on whether measurements were taken from distinct samples or whether the same sample was measured repeatedly
☐	☒	The statistical test(s) used AND whether they are one- or two-sided<br>*Only common tests should be described solely by name; describe more complex techniques in the Methods section.*
☐	☒	A description of all covariates tested
☒	☐	A description of any assumptions or corrections, such as tests of normality and adjustment for multiple comparisons
☐	☒	A full description of the statistical parameters including central tendency (e.g. means) or other basic estimates (e.g. regression coefficient) AND variation (e.g. standard deviation) or associated estimates of uncertainty (e.g. confidence intervals)
☐	☒	For null hypothesis testing, the test statistic (e.g. *F*, *t*, *r*) with confidence intervals, effect sizes, degrees of freedom and *P* value noted<br>*Give P values as exact values whenever suitable.*
☒	☐	For Bayesian analysis, information on the choice of priors and Markov chain Monte Carlo settings
☐	☒	For hierarchical and complex designs, identification of the appropriate level for tests and full reporting of outcomes
☒	☐	Estimates of effect sizes (e.g. Cohen's *d*, Pearson's *r*), indicating how they were calculated

Our web collection on statistics for biologists contains articles on many of the points above.

Software and code

Policy information about availability of computer code

Data collection	We used a medical imaging diagnosis support software, version 3.5.1 (Huiying Medical Technology Co. LTD, Beijing) to collect the volume and the ratios of the lesions in bilateral lungs of the chest CT images.
Data analysis	All analyses were conducted with SAS software, version 9.4 (SAS Institute, USA).

For manuscripts utilizing custom algorithms or software that are central to the research but not yet described in published literature, software must be made available to editors and reviewers. We strongly encourage code deposition in a community repository (e.g. GitHub). See the Nature Research guidelines for submitting code & software for further information.

Data

Policy information about availability of data

All manuscripts must include a data availability statement. This statement should provide the following information, where applicable:

- Accession codes, unique identifiers, or web links for publicly available datasets
- A list of figures that have associated raw data
- A description of any restrictions on data availability

Anonymized data used for the analysis in this article are public at https://figshare.com/s/e81cb26a8584964ba469.

Field-specific reporting

Life sciences study design

All studies must disclose on these points even when the disclosure is negative.

Sample size	This is a phase II trial about repurposing drug use to preliminarily explore the efficacy and safety of bevacizumab in Covid-19. Enable to discover promising drug as soon as possible, and avoid investigation of ineffective drug on more patients, as well as consider the limited resources in the pandemic situation, we preset a sample size of 20-30 cases. We did our best and enrolled twenty-six patients. This number is similar to other trials of repurposing use of drug in Covid-19 or bevacizumab in oncology (J Clin Invest 2020;130(12):6409-6416; Lancet Oncol 2017;18(9):1172-1181; and J Clin Oncol 2009; 27(25):4076-81). In addition, we established an external control group and performed case-control analysis.
Data exclusions	There was one patient who withdrew the consent to cooperate the acquisition of observation indicators of outcomes (who recovered well and was discharged from hospital). His safety data were included in adverse events analysis.
Replication	This study represents the first in-human clinical trial about the effect of bevacizumab on Covid-19, no replication is performed in this study. We have the protocol with the manuscript to ensure the reproducibility of this study.
Randomization	This is not a randomized study. Owing to the pandemic situation in Wuhan and Lombardia during February and March of 2020, which were the most heavily affected areas around the globe, investigators in both centers had limited resources, medical devices, and stressful working environment to conduct a randomized controlled trial. We used two comparisons to investigate the efficacy and control the covariates, 1) self-control, a comparison of pre- and post- treatment measures; 2) external control, a comparison to untreated controls.
Blinding	The blinding of group allocation is not relevant to our study because it is a single-arm study. The data analysis is not blinding because the statisticians know the intervention due to the single-arm design. But during the analysis, we avoid the involvement of investigators as much as possible.

Behavioural & social sciences study design

All studies must disclose on these points even when the disclosure is negative.

Study description	*Briefly describe the study type including whether data are quantitative, qualitative, or mixed-methods (e.g. qualitative cross-sectional, quantitative experimental, mixed-methods case study).*
Research sample	*State the research sample (e.g. Harvard university undergraduates, villagers in rural India) and provide relevant demographic information (e.g. age, sex) and indicate whether the sample is representative. Provide a rationale for the study sample chosen. For studies involving existing datasets, please describe the dataset and source.*
Sampling strategy	*Describe the sampling procedure (e.g. random, snowball, stratified, convenience). Describe the statistical methods that were used to predetermine sample size OR if no sample-size calculation was performed, describe how sample sizes were chosen and provide a rationale for why these sample sizes are sufficient. For qualitative data, please indicate whether data saturation was considered, and what criteria were used to decide that no further sampling was needed.*
Data collection	*Provide details about the data collection procedure, including the instruments or devices used to record the data (e.g. pen and paper, computer, eye tracker, video or audio equipment) whether anyone was present besides the participant(s) and the researcher, and whether the researcher was blind to experimental condition and/or the study hypothesis during data collection.*
Timing	*Indicate the start and stop dates of data collection. If there is a gap between collection periods, state the dates for each sample cohort.*
Data exclusions	*If no data were excluded from the analyses, state so OR if data were excluded, provide the exact number of exclusions and the rationale behind them, indicating whether exclusion criteria were pre-established.*
Non-participation	*State how many participants dropped out/declined participation and the reason(s) given OR provide response rate OR state that no participants dropped out/declined participation.*
Randomization	*If participants were not allocated into experimental groups, state so OR describe how participants were allocated to groups, and if allocation was not random, describe how covariates were controlled.*

Ecological, evolutionary & environmental sciences study design

All studies must disclose on these points even when the disclosure is negative.

Study description	*Briefly describe the study. For quantitative data include treatment factors and interactions, design structure (e.g. factorial, nested, hierarchical), nature and number of experimental units and replicates.*
Research sample	*Describe the research sample (e.g. a group of tagged Passer domesticus, all Stenocereus thurberi within Organ Pipe Cactus National Monument), and provide a rationale for the sample choice. When relevant, describe the organism taxa, source, sex, age range and any manipulations. State what population the sample is meant to represent when applicable. For studies involving existing datasets,*

describe the data and its source.

Sampling strategy	Note the sampling procedure. Describe the statistical methods that were used to predetermine sample size OR if no sample-size calculation was performed, describe how sample sizes were chosen and provide a rationale for why these sample sizes are sufficient.
Data collection	Describe the data collection procedure, including who recorded the data and how.
Timing and spatial scale	Indicate the start and stop dates of data collection, noting the frequency and periodicity of sampling and providing a rationale for these choices. If there is a gap between collection periods, state the dates for each sample cohort. Specify the spatial scale from which the data are taken
Data exclusions	If no data were excluded from the analyses, state so OR if data were excluded, describe the exclusions and the rationale behind them, indicating whether exclusion criteria were pre-established.
Reproducibility	Describe the measures taken to verify the reproducibility of experimental findings. For each experiment, note whether any attempts to repeat the experiment failed OR state that all attempts to repeat the experiment were successful.
Randomization	Describe how samples/organisms/participants were allocated into groups. If allocation was not random, describe how covariates were controlled. If this is not relevant to your study, explain why.
Blinding	Describe the extent of blinding used during data acquisition and analysis. If blinding was not possible, describe why OR explain why blinding was not relevant to your study.

Did the study involve field work? ☐ Yes ☐ No

Field work, collection and transport

Field conditions	Describe the study conditions for field work, providing relevant parameters (e.g. temperature, rainfall).
Location	State the location of the sampling or experiment, providing relevant parameters (e.g. latitude and longitude, elevation, water depth).
Access & import/export	Describe the efforts you have made to access habitats and to collect and import/export your samples in a responsible manner and in compliance with local, national and international laws, noting any permits that were obtained (give the name of the issuing authority, the date of issue, and any identifying information).
Disturbance	Describe any disturbance caused by the study and how it was minimized.

Reporting for specific materials, systems and methods

We require information from authors about some types of materials, experimental systems and methods used in many studies. Here, indicate whether each material, system or method listed is relevant to your study. If you are not sure if a list item applies to your research, read the appropriate section before selecting a response.

Materials & experimental systems

n/a	Involved in the study	
☒	☐	Antibodies
☒	☐	Eukaryotic cell lines
☒	☐	Palaeontology and archaeology
☒	☐	Animals and other organisms
☐	☒	Human research participants
☐	☒	Clinical data
☒	☐	Dual use research of concern

Methods

n/a	Involved in the study	
☒	☐	ChIP-seq
☒	☐	Flow cytometry
☒	☐	MRI-based neuroimaging

Human research participants

Policy information about studies involving human research participants

Population characteristics	Severe Covid-19 patients with respiratory rate ≥30 times/min, oxygen saturation ≤93% with ambient air, or partial arterial oxygen pressure to fraction of inspiration O2 ratio (PaO2/FiO2) >100mmHg and ≤300 mmHg, and diffuse pneumonia confirmed by chest radiological imaging, were included. The median age of patients was 62 years, and 20 (77%) were men. Thirteen patients had hypertension history (50%) and six (23%) had diabetes history. Other detailed baseline characteristics are shown in Table 1.
Recruitment	All eligible patients with Covid-19 were screened by investigators in the two centers. After the the informed consent of patients, the investigators carefully evaluated the inclusion and exclusion criteria as shown in Methods to confirm the inclusion of patients. Controls were included retrospectively who were hospitalized in the similar time frame in the same centers. Selection bias exist in external controls, because the controls were collected retrospectively and not randomized. This leads to a decreased statistical power of comparative analysis with the control.
Ethics oversight	The trial was approved by the ethics committees of Qilu Hospital of Shandong University, Renmin Hospital of Wuhan University, and Italia Hospital S.p.A. Ospedale Generale di Zona Moriggia - Pelascini, Gravedona ed Uniti (CO).

Note that full information on the approval of the study protocol must also be provided in the manuscript.

Clinical data

Policy information about clinical studies

All manuscripts should comply with the ICMJEguidelines for publication of clinical research and a completedCONSORT checklist must be included with all submissions.

Clinical trial registration	ClinicalTrials.gov Identifier: NCT04275414
Study protocol	The study protocol is available in supplementary files.
Data collection	Patients were enrolled in Renmin Hospital of Wuhan University, Wuhan, Hubei Province, China from February 15 to March 8, 2020, and in Italia Hospital S.p.A. Ospedale Generale di Zona Moriggia - Pelascini, Gravedona ed Uniti (CO), Italy from March 25 to April 5, 2020, and followed up for 28 days or until hospital discharge. Data were collected during the above period. The external controls were hospitalized in the same center within the similar timeframe (±5 days), i.e., between the 10th of February and the 13th of March of 2020 in China and between the 20th of March and the 10th of the April of 2020 in Italy. Data of controls were collected retrospectively in china from March 10 to April 2, and in Italy from April 20 to May 8.
Outcomes	Primary outcome: Partial arterial oxygen pressure (PaO2) to fraction of inspiration O2 (FiO2) ratio [Time Frame: 24 hours] and [Time Frame: 7 days]. PaO2/FiO2 are measured by arterial blood gas analysis.<br>Main secondary outcomes:<br>(1) Rate of improvement of oxygen-support status [Time Frame: 28 days]. The oxygen-support status includes 6 levels: mechanical ventilation, non-invasive ventilation, a transition status of alternate use of non-invasive ventilation and high-flow oxygen, high-flow oxygen, low-flow oxygen and ambient air. The improvement of oxygen-support status is defined as switch from a higher level of oxygen-support to a lower level.<br>(2) The change of areas of pulmonary lesions shown on chest radiological imaging (chest CT or X-ray) [Time Frame: 7 days]. The areas of pulmonary lesions are analyzed by professional imaging software.
